# Current Management Options in Irvine–Gass Syndrome: A Systemized Review

**DOI:** 10.3390/jcm10194375

**Published:** 2021-09-25

**Authors:** Michał Orski, Maciej Gawęcki

**Affiliations:** 1 Department of Ophthalmology, Ludwik Rydygier Memorial Hospital, Zlotej Jesieni 1, 31-826 Krakow, Poland; 2 Dobry Wzrok Ophthalmological Clinic, Kliniczna 1B/2, 80-402 Gdansk, Poland; maciej@gawecki.com

**Keywords:** Irvine–Gass syndrome, cystoid macular edema, pseudophakic cystoid macular edema, NSAIDs corticosteroids, anti-VEGF, subthreshold diode micropulse

## Abstract

Irvine–Gass syndrome (IGS) remains one of the most common complications following uneventful cataract surgery. In most cases, macular edema (ME) in IGS is benign, self-limiting, and resolves spontaneously without visual impairment; however, persistent edema and refractory cases may occur and potentially deteriorate visual function. Despite the relatively high prevalence of IGS, no solid management guidelines exist. We searched the PUBMED database for randomized clinical trials (RCT) or case series of at least 10 cases published since 2000 evaluating different treatment strategies in patients with cystoid macular edema (CME). The search revealed 28 papers that fulfilled the inclusion criteria with only seven RCTs. The scarceness of material makes it impossible to formulate strong recommendations for the treatment of IGS. Clinical practice and theoretical background support topical non-steroidal anti-inflammatory drugs (NSAIDs) as the first-line therapy. Invasive procedures, such as periocular steroids, intravitreal corticosteroids, and anti-vascular endothelial growth factor (anti-VEGF), are usually applied in prolonged or refractory cases. Results of novel applications of subthreshold micropulse laser (SML) are also promising and should be studied carefully in terms of the safety profile and cost effectiveness. Early initiation of invasive treatment for providing better functional results must be examined in further research.

## 1. Introduction

Postoperative cystoid macular edema (CME) remains one of the most common complications of intraocular surgery. It is defined as a presence of intraretinal fluid (IF) spaces or central macular thickening (CMF) in optical coherence tomography (OCT) examination [1]. Irvine–Gass syndrome (IGS), sometimes named pseudophakic cystoid macular edema (PCME), is a cystoid macular edema that develops following uneventful cataract surgery. It was first described in 1953 by Irvine and studied using fluorescein angiography (FA) by Gass and Norton in 1966 [2,3]. Irvine–Gass syndrome remains the most common cause of decreased visual acuity after uneventful cataract surgery [4]. In most cases, no treatment is indicated as it resolves spontaneously, but persistent edema may also occur. Hunter et al. reported that 26.8% of eyes with pseudophakic CME did not recover 6/6 vision [5].

The incidence of Irvine–Gass syndrome varies among studies and is highly dependent on the diagnostic criteria [6]. Diagnosis is made based on clinical findings along with visual impairment or based on the presence of FA leakage or IF on OCT scans. OCT shows cystic intraretinal spaces on high-resolution cross-sectional scans of the macula that can be accompanied by mild photoreceptors detachment [4,7]. The early phases of FA show macular leakage, and as FA helps to rule out other causes of macular edema (ME), it remains a gold standard as a diagnostic tool [8] when used with the OCT.

Clinically significant CME impairing patients’ vision is found in 1–2% of patients with its peak 6 weeks following surgery, but subclinical CME can be seen in about 30% of patients in FA and up to 40% in OCT [4,7,9]. The risk factors include the presence of epiretinal membrane, history of uveitis, diabetes mellitus, and use of topical medications for glaucoma.

Several models have been considered, but multifactorial inflammatory origin seems to play a major role in the pathophysiology of Irvine–Gass syndrome. Surgical manipulation causes significant release of inflammatory mediators, including arachidonic acid, cytokines, lysozyme, and vascular endothelial growth factor (VEGF). The inflammatory cascade impairs the blood–aqueous and blood–retinal barriers and promotes vascular permeability [10,11]. Fluid accumulates in the outer plexiform and inner nuclear layers, creating cystic intraretinal spaces that coalesce to larger fluid cavities [6]. Prolonged CME may cause lamellar holes and persistent subretinal fluid.

To date, there are no uniform recommendations for the treatment of Irvine–Gass syndrome, and variable strategies are employed. This review aims to present the most important contemporary therapeutic strategies in IGS based on available modern literature.

## 2. Material and Methods

The PUBMED database was searched for a combination of phrases including the terms Irvin–Gass syndrome or pseudophakic cystoid edema and steroids, intravitreal steroids, periocular steroids, triamcinolone, sub-tenon triamcinolone, dexamethasone, OZURDEX^®^, fluocinolone, non-steroidal anti-inflammatory drugs, anti-VEGF, aflibercept, ranibizumab, bevacizumab, carbonic anhydrase inhibitors, and acetazolamide.

Only randomized clinical trials or case series of at least 10 cases published since 2000 were included in the analysis and presented in the following tables. Reports using smaller samples were quoted only if larger studies were scarce or unavailable for the specific treatment modality.

The search revealed 28 articles, including 7 RCTs on the subject, that fulfilled inclusion criteria. Results were grouped according to the analyzed treatment modality.

If a treatment modality was not analyzed in a larger case series or RCT, results were presented descriptively.

## 3. Results

### 3.1. Non-Steroidal Anti-Inflammatory Drugs (NSAID)

The search revealed seven studies, including two RCTs, that met inclusion criteria analyzing the efficacy of NSAID eye drops in the treatment of IGS. The results of those studies are presented in Table 1. All the studies show functional and morphological improvement, although most patients still present some visual deficit at the end of the treatment. The latest studies favor topical nepafenac compared to other NSAID eye drops. No significant adverse events associated with the use of NSAIDs were reported in any of the studies.

### 3.2. Carbonic Anhydrase Inhibitors (CAI)

The search revealed only 2 studies that analyzed the additional effect of 250–500 mg of oral acetazolamide compared to that from topical NSAIDs or corticosteroids alone (Table 2). Both papers present better functional and morphological results of combined NSAID with or without corticosteroid plus CAI. Both papers present better functional and morphological results of NSAID combined with CAI. No data evaluating the potential role of topical CAIs were found.

### 3.3. Corticosteroids

#### 3.3.1. Topical Corticosteroids

The search revealed only two studies that analyzed the additional effect of topical corticosteroids compared to NSAIDs alone. The results are presented in Table 3 and do not provide an unequivocal answer whether any additional effect exists: possible benefits are advocated in the Heier et al. study [21] but not confirmed in the study by Singal et al. [22].

#### 3.3.2. Periocular Corticosteroids

The search revealed only three papers fulfilling the search criteria. The results of these studies are presented in Table 4. All are retrospective analyses and present significant improvement of both macular morphology and BCVA after sub-tenon injection of triamcinolone acetonide (STT) in IGS patients. A study by Kuley et al. [23] compared the effects of STT and IVT in a large sample but did not show a significant difference in final effect depending on the drug administration route.

#### 3.3.3. Intravitreal Corticosteroid

In the search, we found eight larger reports published since 2000^t^ that are presented in Table 5. The search revealed only two larger studies evaluating the efficacy of IVT in IGS (listed in Table 5). However, randomized controlled trials of IVT are missing. In addition, transient effects and the need for repeated injections remain a challenge [26]. Most high-quality studies on the use of intravitreal corticosteroids in IGS are focused on the use of an intravitreal dexamethasone implant (IVD) of 700 micrograms, commercially used under the name OZURDEX^®^ (five studies). One study associated the results of IGS treatment with either IVD, IVT, or anti-VEGF to the time point of initiation of treatment [27]. Most of the patients were treated with IVD. All the listed studies demonstrated significant letter gains after intravitreal corticosteroid therapy without serious adverse events. Few cases of intraocular pressure rise were controlled with topical anti-glaucoma medication. The study by Sharma and his group showed that early initiation of intravitreal treatment in IGS provides better functional results [27]. The use of the a fluocinolone implant was not tested on a larger sample; however, available reports confirm its efficacy in the resolution of IGS in recurrent cases [28].

### 3.4. Anti-VEGF

The search revealed six larger studies analyzing the effects of different anti-VEGF medications in the treatment of IGS: four studies employed intravitreal bevacizumab (IVB), one dedicated to intravitreal ranibizumab (IVR) and one compared the efficacy of the available three agents: aflibercept, ranibizumab, and bevacizumab. Results of those studies are presented in Table 6 and show significant visual and morphological improvements for all the available anti-VEGF medications without serious adverse effects. Intravitreal aflibercept (IVA), a more recent anti-VEGF agent, has been tried in the treatment of IGS, but except for one comparative study listed in Table 6, only case reports have been published on the use of aflibercept [36].

Anecdotal reports of combined intravitreal anti-VEGF and corticosteroids in the treatment of IGS exist, but these are only case reports, not larger trials [37]. Therefore, it is difficult to judge the additional effect of those drugs compared to anti-VEGF therapy alone in IGS.

### 3.5. Subthreshold Micropulse Laser (SML)

A photostimulation process with repetitive short pulses delivered at a subthreshold mode allows foveal treatment with no damage compared to conventional laser treatments. The benefits of SML in the treatment of different macular disorders such as central serous chorioretinopathy (CSC), diabetic macular edema (DME), and macular edema secondary to retinal vein occlusion (RVO) were shown in many studies [44,45].

In 2020, Verdina et al. published the first results of the treatment of refractory postoperative CME with subthreshold micropulse yellow laser in 10 eyes of 10 patients [46]. Five eyes of five patients had Irvine–Gass syndrome. A retrospective analysis showed improvement of BCVA and CMT in all patients, and the effects were maintained through 1, 2, 3, and 6 months. The treatment used a 577 nm subliminal laser photo-stimulation treatment with 7 × 7 grids with confluent spots and a 5% duty cycle. Treatment was targeted at whole edematous retina, including the foveal center. The study demonstrated complete resolution of retinal edema and improvement of BCVA in all patients with no side effects. The mean number of laser treatments was 1.3.

### 3.6. Laser Photocoagulation (LPC)

No studies of LPC in IGS published after 2000 were found in the PUBMED database. Previous studies reported a beneficial effect of modified GRID protocol for IGS; however, these were not controlled studies [47].

### 3.7. Other Treatments

Interferon alfa was administered for IGS in a small case series of four eyes refractory to topical treatment [48]. A 3 million IU/day dose was injected subcutaneously for 4 weeks and tapered thereafter. Improvement was achieved in three cases without any side effects. Topical treatment of chronic refractory IGS with interferon alfa was also reported in a single case with spectacular visual improvement from 20/100 to 20/25 [49].

IGS was also treated by adalimumab (Humira). No significant improvement after such therapy was achieved in a small case series of five eyes [50].

## 4. Discussion

The excellent results of modern cataract surgery set patient expectations very high, and persistent CME after uneventful cataract surgery may significantly affect patient outcomes and satisfaction [51]. Irvine–Gass syndrome is a common complication of uneventful cataract surgery, which resolves spontaneously in most cases but may persist, causing visual deterioration and patient dissatisfaction [4,6,7]. As has been emphasized in many previous reviews and studies, no homogenous recommendations for the treatment of IVG exist [52,53,54,55]. The lack of randomized controlled trials assessing the effectiveness of available therapeutic modalities results in many different approaches, often based on individual judgment and clinical experience but not hard evidence. Our analysis focused on the papers published in this century, as this is the time when intravitreal treatments such as anti-VEGF or intravitreal corticosteroids were introduced and revolutionized the management of various ophthalmic diseases. Therefore, we sought to compare conservative treatments to those modern therapeutic modalities.

Presented studies published since 2000^t^ in general show favorable results of the treatment of IGS with topical NSAIDs alone or in combination with periocular or intravitreal steroids as well as intravitreal anti-VEGF agents. Those treatments should be considered, weighing both the potential for improving BCVA and the invasive character of the treatment and the possibility of complications.

As IGS resolves spontaneously in most cases, that possibility must be considered before administering invasive therapy. Therefore, the timing of the application of different forms of treatment should be carefully considered with non-invasive therapies used as the first line (e.g., topical treatment) and invasive procedures (e.g., intraocular injections) usually reserved for non-responsive cases.

NSAIDs administered topically such as via eye drops are FDA-approved drugs for use as anti-inflammatory, antipyretic, and analgesic agents. Their main mechanism of action is the inhibition of the enzyme cyclooxygenase (COX). Cyclooxygenase is required to convert arachidonic acid into thromboxanes, prostaglandins, and prostacyclin. Prostaglandins play an important role in vasodilatation [56]. The use of NSAIDs in the postoperative management of patients undergoing cataract surgery has become a standard of care [57,58]. Routine use of anti-inflammatory eye drops following cataract surgery is highly effective in reducing post-surgical inflammation and the incidence of CME [59]; however, their role in the treatment of CME has not been studied widely. Topical NSAIDs remain a first-line therapy of IGS, and although their use has shown to be beneficial in several studies, they have shown no clear effect in other studies [58]. Our search revealed only a few modern studies that analyze the effects of NSAID in the treatment of IGS, none of which is an RCT. One older study showed significant visual and morphological improvements after administration of topical NSAID in acute cases, which are usually defined as lasting less than 3 months [18]. However, most recent studies show only moderate improvement after treatment of IGS with only topical NSAID [12,13].

Functional and morphological results are reported to be better after intravitreal dexamethasone [13]. Adding the effect of nepafenac was reported in one study that analyzed the combination of IVB and NSAID [16]. NSAIDs are also used in combined therapy with topical corticosteroids or oral CAIs, but available data on the combined treatment of CME are very limited. Nevertheless, the off-label use of acetazolamide, a carbonic anhydrase inhibitor, in IGS is a common practice as a first- or second-line therapy. Acetazolamide increases the retinal pigment epithelium pump function by inhibiting carbonic anhydrase and is thought to decrease intraretinal fluid [11,60]. Dosage varies among studies from 250 mg once a day to TID. Many authors state that the combination of oral acetazolamide with topical NSAIDs is shown to be highly effective [20,61,62]. Our search revealed only two papers analyzing the additional effect of CAI compared to NSAID-only treatment of IGS, both on relatively small samples (14–15 eyes). Both papers favor the use of CAI in combination therapy for IGS; however, such limited data make it impossible to build strong recommendations for the use of this treatment regimen.

Corticosteroids remain a viable therapeutic option in the treatment of CME, including IGS. Corticosteroids block the release of arachidonic acid, impact the production of interleukins and VEGF, and interrupt the inflammatory cascade. Several routes of administration, such as topical, periocular, and intravitreal, are available. At the same time, current data on a combination treatment of topical NSAID with topical corticosteroids are scarce and not convincing [21,22]. A conclusion on the beneficial effect of the addition of topical corticosteroids to the treatment of IGS cannot be made based on available research. Nevertheless, topical corticosteroids are widely used in the treatment of Irvine–Gass syndrome, usually in combination with topical NSAIDs and oral CAIs. An accurate assessment of the role of topical corticosteroids alone in the treatment of IGS is not currently possible.

Periocular or intravitreal corticosteroids serve as an option in refractory cases of IGS [63]. Sub-tenon or retrobulbar injections of corticosteroids had been used widely for persistent CME before the advent of an officially registered intravitreal dexamethasone implant (OZURDEX^®^). Early in 1997, Thach and his group showed VA improvement after 12 repeated corticosteroid injections in a series of 31 patients with chronic CME [64]. Our search revealed three recent studies (2018–2021) that showed significant visual improvement after STT in refractory cases of IGS. STT remains a cost-effective therapy, and its application sub-tenon does not bear the risk of intraocular inflammation possible after intravitreal application. A recent study by Kuley did not show an advantage of intravitreal versus sub-tenon administration of triamcinolone [23]. It must be emphasized, though, that the use of triamcinolone acetonide remains off-label. Intravitreal corticosteroids have consequently been used for chronic or refractory cases, lasting longer than 3 months, with significant letter gains and minor adverse effects [31,32,33,34,35]. Before the dexamethasone implant was introduced, triamcinolone acetonide was tested in a few larger and smaller studies, proving its efficacy in improving macular morphology and function in IGS [33,35,65,66,67]. Later studies show significant improvements after IVD administration without serious side effects [27,29,30,31,32,34]. The most recent large retrospective study from 2020 highlighted the benefits of early intervention and reported significantly larger visual gains when IVD was administered within 4 weeks of diagnosis [27]. This approach is not a common practice due to the invasive character of the procedure and the possibility of effective treatment with only topical NSAIDs. Further comparative studies are needed to support the results of that paper.

Vascular endothelial growth factors play central roles in the regulation of angiogenesis and lymphangiogenesis and they regulate endothelial cell proliferation, migration, vascular permeability, secretion, and other endothelial functions. The revolutionary role of anti-VEGF in treating ophthalmic conditions such as neovascularization and macular edema due to DME or ME in RVO was a milestone. The VEGF family plays a major role in angiogenesis, inflammation, and capillary permeability; thus, its potential in treating CME was studied. However, the role of anti-VEGF treatment in CME remains unclear. Anti-VEGF injections remain an alternative in unresponsive cases, but their use in IGS requires further randomized research. Our search revealed a few quality studies that show significant improvements after the use of anti-VEGF medication in IGS, but RCTs are missing. Despite that, clinical practice and the universality of that procedure make it a solid treatment modality in refractory IGS.

Non-damaging laser therapy, such as SML, remains an interesting therapeutic option. To date, just a few papers report its efficacy in IGS. Considering its non-damaging character, lack of side effects, and low cost, it may be considered as an alternative to more invasive treatment modalities. Further studies are needed to provide treatment guidelines for SML.

### Practical Considerations and Conclusions

This review aimed to provide a basis for modern recommendations for treating pseudophakic macular edema or Irvine–Gass syndrome. The available published material does not provide convincing data to build such guidelines. Therefore, theoretical background, clinical experience, and safety of the procedure must determine the choice of treatment in this clinical entity. Common practice is to start therapy with a topical NSAID, which is a simple and non-invasive treatment modality. This approach is supported by epidemiological and clinical research that provides data on the possibility of spontaneous resolution of CME and improvement after topical therapy [68]. Larger clinical trials have not shown that using a combination of topical NSAID and topical corticosteroids and/or oral CAI is superior to topical NSAID alone.

What remains unclear is the timing of application of invasive therapies—periocular or intravitreal injections—once topical treatment is not effective. Refractory pseudophakic macular edema is not precisely defined according to its duration, but usually authors employ periocular or intravitreal treatment in cases lasting longer than three months. The efficacy and safety of intravitreal or periocular injections with corticosteroids or anti-VEGF agents have been confirmed in many studies. Still, its invasive nature and rare but potentially serious complications must be considered. Patients who resist intravitreal or periocular treatment might be offered therapy with subthreshold micropulse laser. Recent publications on the use of SML show promise. Low complication rates, cost-effectiveness, and repeatability are clear advantages of this treatment modality.

Our search revealed publications that show possible options for the treatment of IGS. Methodology and randomization in presented trials may be discussed; what remains as their common feature is the visual deficit reported in most cases of longstanding CME, even after successful treatment. Therefore, in view of results of a recent large study from Sharma et al. [27] that proves better functional and morphological results with early application of intravitreal steroids, that therapeutic option for short-standing pseudophakic CME should be examined with care in future research.

## Figures and Tables

**Table 1:** Results of the studies analyzing the efficacy of NSAID in the treatment of IGS that involved at least 10 cases and were published since 2000.

No	Study	No of Eyes	Duration of CME	Study Design	Results
1	Giarmoukakis et al., 2020 [12]	21 eyes treated with TN 0.3%	Acute (<4 months) and chronic (>4 months)	Prospective, clinic-based, non-randomized case-series	BCVA improvement from 0.49 ± 0.36 logMAR to 0.36 ± 0.42 logMAR at the last follow-up visit (*P* < 0.005). CRT decreased from 450.40 ± 90.74 μm at baseline to 354.60 ± 81.49 μm (*P* < 0.05)
2	Guclu et al., 2019 [13]	62 The IVD group included 32 eyes, and the TN group included 30 eyes	2 months	Retrospective; two arms: IVD: 32 patients, TN 0.1%: 30 patients; changes in BCVA, CMT at baseline, 1 month, 3 months, 6 months	Results at 6 months: BCVA change in ETDRS letters for IVD from 25 ± 11.8 to 49.3 ± 6.8 versus 20.9 ± 9.3 to 32.9 ± 7.3 for TN; CMT reduction from 522.7 ± 120.7 μm to 266.1 ± 53.4 μm for IVD versus 501.2 ± 104.2 μm to 364.9 ± 56.3 μm) for TN; statistically significantly better improvements for IVD than TN
3	Sengupta et al., 2018 [14]	69	Acute, precise duratio not defined	Retrospective; combined topical prednisolone QID for 6 weeks and TN for at least 6 weeks QID; evaluation of effect at 6 weeks; success criterion: BCVA 6/9 and CMT £300 mm; definition of any success: anything less than success and reduction of CMT by 150 mm	Success achieved in 37 eyes (54%) and any success in 55 eyes (80%) at 6 weeks
4	Yuksel et al., 2017 [15]	24 TA arm 24 TN arm	Mean duration 4.8 ± 5.0 weeks for TA and 4.5 ± 3.1 weeks for TN	Prospective; two arms: TA and TN; changes in CMT and BCVA at 6 months	Significant reduction of CMT and improvement of BCVA in both groups; BCVA change from 0.99 ± 0.62 logMAR to 0.63 ± 0.74 for TA and from 0.84 ± 0.65 to 0.37 ± 0.48 for TN reduction of CMT from 513.3 to 318.9 mm in TA arm and from 483.7 to 278.0 mm in TN arm; BCVA statistically better improvement in the TN arm
5	Warren et al., 2010 [16]	39	Chronic 6 months, mean 9.4 months	RCT; evaluation of the effect of adding topical NSAID in IGS; Design: IVT and IVB at study entry; IVB repeated after 1 month; afterward randomization to topical diclofenac 0.1% or ketorolac 0.4% or nepafenac 0.1% or bromfenac 0.09% or placebo for 16 weeks; evaluation at 16 weeks	Significant reduction of CMT compared with placebo for TN and topical bromfenac; improvement of BCVA for nepafenac only (by 19%)
6	Hariprasad et al., 2009 [17]	22 eyes with pseudophakic and uveitic CME, including 13 with chronic IGS and 3 with acute IGS (20 patients) treated with TN 0.1%)	Acute IGS < 6 months Chronic IGS > 6 months	Retrospective multicenter review of 22 CME cases treated with TN 0.1% (six with concomitant prednisolone acetate 1%); duration of the follow up from 6 weeks to 6 months	BCVA improvement in 2 acute IGS (from 0.4 logMAR to 0.18 logMAR and from 0.3 logMAR to 0.14 logMAR). CRT reduction from 448 to 211 mm and from 306 to 284 mm. Morphological improvement in the third acute case: reduction of CMT from 380 to 236 mm, but no BCVA change due to retinal degeneration; mean BCVA improvement in the chronic group from 0.63 ± 0.33 logMAR to 0.30 ± 0.16 logMAR and mean CMT reduction from 451 ± 145.7 to 273 ± 80.8 mm
7	Rho 2003 [18]	34: Diclofenac 18 Ketorolac 16	Acute: 4.2 ± 1.4 months for ketorolac group and 4.0 ± 1.4 months for diclofenac group	Randomized prospective; evaluation of effects of topical diclofenac sodium 0.1% versus ketorolac tromethamine 0.5% in the treatment of IGS; evaluation at 26 weeks	BCVA change ketorolac: from 20/160 ± 75.8 to 20/58 ± 94.1 diclofenac: from 20/173 ± 94 to 20/49 ± 56.8 Reduction of CME at 26 weeks: diclofenac 16 (89%), ketorolac 14 (88%); elimination of CME at 26 weeks: diclofenac 14 (78%), ketorolac 12 (75%); no significant difference between the drugs

RCT: randomized controlled trial; IGS: Irvine–Gass syndrome; ME: macular edema; IVD: intravitreal dexamethasone implant; FA: fluorescein angiography; IVB: intravitreal bevacizumab; CMT: central macular thickness; TA: triamcinolone acetonide; TN: topical nepafenac; BCVA: best-corrected visual acuity; CME: cystoid macular edema; QID–quater in die.

**Table 2:** Results of the studies analyzing the efficacy of CAI in the treatment of IGS that involved at least 10 cases and were published since 2000.

No	Study	No of Eyes	Duration of CME	Study Design	Results
1	Curkovic et al., 2005 [19]	14 7–0.1% topical dexamethason + topical flurbiprofen (group 1) 7–0.1% topical dexamethason + topical flurbiprofen plus acetazolamide 250 mg 3× (group 2)	Not defined	RCT, the efficacy of oral acetazolamide of 250 mg TID in addition to topical dexamethasone and flurbiprofen	Complete resolution of CME in 86% of eyes receiving acetazolamide (plus the topical NSAID-steroid combination) vs. 29% in the control group who received topical dexamethasone and flurbiprofen alone BCVA change significantly better in group 2 from 0.32 ± 0.1 to 0.67 ± 0.1 versus 0.34 ± 0.12 to 0.53 ± 0.14 in group 1 (Snellen fraction)
2	Catier et al., 2005 [20]	16	5 months	Retrospective review 250–500 mg of acetazolamide per day associated with topical NSAID or steroids	Mean improvement of BCVA from 20/100 (0.7 ± 0.28 Log MAR) to 20/40 (+0.3 ± 0.2 Log MAR) and reduction of CMT from 599.67 ± 174.17 mm to 264.69 ± 106.59 mm; complete resolution in 87.5% cases and in 100% of cases treated by a combination of acetazolamide, NSAIDs and steroids

CT: randomized controlled trial; IGS: Irvine–Gass syndrome; CMT: central macular thickness; NSAID: non-steroidal anti-inflammatory drugs; BCVA: best-corrected visual acuity; CME: cystoid macular edema; mo—month; TID—ter in die.

**Table 3:** Results of the studies analyzing the efficacy of the addition of topical corticosteroids to NSAID in the treatment of IGS that involved at least 10 cases and were published since 2000.

No	Study	No of Eyes	Duration of CME	Study Design	Results
1	Heier et al., 2000 [21]	28 (26 completed the study)	Acute: 21–90 days after surgery	RCT, patients randomized to topical therapy with ketorolac (group K), prednisolone (group P), or ketorolac and prednisolone combination therapy (group C) QID. Follow up, 3 months.	BCVA improvements (Snellen lines): 1.6 in group K, 1.21 in group P, and 3.8 in group C. Treatment of acute, visually significant pseudophakic CME with ketorolac and prednisolone combination therapy appears to offer benefits over monotherapy with either agent alone
2	Singal et al., 2004 [22]	10 Ketorolac: 4 Ketorolac and tromethamine: 6	6 weeks and longer	RCT: prospective double-masked randomized controlled trial. 10 patients were randomly assigned to receive either 0.5% ketorolac tromethamine plus placebo or 0.5% ketorolac tromethamine plus 1% prednisolone acetate; follow up, 90 days	No statistically significant difference was found in the outcome between patients who received ketorolac and those who received ketorolac plus prednisolone for acute or chronic CME

RCT: randomized controlled trial; IGS: Irvine–Gass syndrome; CMT: central macular thickness NSAID: non-steroidal anti-inflammatory drugs; BCVA: best-corrected visual acuity; CME: cystoid macular edema.

**Table 4:** Results of the studies analyzing the efficacy of periocular corticosteroids in the treatment of IGS that involved at least 10 cases and were published since 2000.

No	Study	No of Eyes	Duration of CME	Study Design	Results
1	Kuley et al., 2021 [23]	50 STT 45 IVT	Not stated	Retrospective; comparison of resolution of IGS in two arms: 2 mg IVT or 40 mg STT at 1, 3, and 6 months	Insignificant difference in BCVA improvement: 2.3 lines in the IVT group and 2.4 lines in the STT group; CMT reduction was significantly better in the IVT group at month 1 (255 mm vs. 187 mm), but the difference was not present at month 3 (214 mm vs. 212 mm) and month 6 (176 mm vs. 207 mm); ocular hypertension managed by topical therapy in 7% of eyes in the IVT group and 12% of eyes in the STT group
2	Erden et al., 2019 [24]	21	Not stated	Retrospective; patients treatment naïve; injection of 40 mg of STT; minimum follow up 6 months	Significant improvement of mean BCVA from 0.71 ± 0.23 logMAR to 0.19 ± 0.06 logMAR and significant reduction of CMT from 431 ± 136 mm to 299 ± 66 mm at 6 months
3	Tsai et al., 2018 [25]	17	57.9 ± 50.1 days (range: 21–178 days).	Retrospective; 40 mg of STT; evaluation of BCVA and CMT at 1 and 3 months	Change of logMAR BCVA from baseline 0.75 ± 0.23 to 0.50 ± 0.20 at month 1 and 0.40 ± 0.20 at month 3. Change of CMT from baseline 446 ± 107 mm to 354 ± 90 mm at month 1 and 300 ± 58 mm at month 3. Insignificant rise of IOP < 21 mm Hg

RCT: randomized controlled trial; IGS: Irvine–Gass syndrome; ME: macular edema; IVD: intravitreal dexamethasone implant; FA: fluorescein angiography; IVB: intravitreal bevacizumab; IVT—intravitreal triamcinolone; CMT: central macular thickness; TA: triamcinolone acetonide; STT: sub-tenon triamcinolone; IOP: intraocular pressure; BCVA: best-corrected visual acuity.

**Table 5:** Results of the studies analyzing the efficacy of intravitreal corticosteroids in the treatment of IGS that involved at least 10 cases and were published since 2000.

No	Study	No of Eyes	Duration of CME (Months)	Study Design	Results
1	Sharma et al., 2020 [27]	79	Less than 14 weeks	Retrospective; evaluation of the effect of IVD or IVT or anti-VEGF in IGS; evaluation at 12 months	IVD in 73.4% of eyes as initial therapy; switch from anti-VEGF to dexamethasone in 54.5% of cases; BCVA gain and CMT reduction 16.7 ± 12.9 letters and 336.7 ± 191.7 mm in patients treated within 4 weeks from diagnosis versus 5.2 ± 9.2 letters and 160.1 ± 153.1 mm for patients treated after 14 weeks from diagnosis; IOP rise in 3 patients after IVD controlled with topical medications
2	Altintas et al., 2019 [29]	10	Minimum 3 months	Retrospective; IGS resistant to topical treatment and IVB; implantation of IVD	Significant improvement of mean BCVA from 0.69 ± 0.19 logMAR to 0.19 ± 0.05 logMAR and significant reduction of mean CMT from 476.13 ± 135.13 mm to 268.38 ± 31.35 mm; mean number of IVD: 1.44 ± 0.89
3	Bellocq et al., 2017 [30]	100	Mean 4.8 months	Retrospective multicenter national case series of 100 eyes receiving IVD for post-surgical macular edema	Mean improvement in BCVA was 9.6 ± 10.6 letters at month 6 and 10.3 ± 10.7 letters at month 12; BCVA gains of 15 or more letters noted in 32.5% cases and 37.5% cases at months 6 and 12, respectively; mean reduction in CSMT of 135.2 mm and 160.9 mm at months 6 and 12, respectively 37% of patients required only one IVD during the first year and experienced no recurrence of the macular edema in a follow-up period of greater than 1 year
4	Mayer et al., 2015 [31]	23	Mean 5.4 months (range 2–8)	Prospective; treatment with IVD; evaluation of BCVA and CMT at 12 months	Significant improvement of mean BCVA from 30.2 ± 4.3 letters to 50.4 ± 4.9 letters and decrease of CMT from 520.8 ± 71.4 mm to 232.7 ± 26.6 mm; no relevant adverse effects were noted
5	Zamil 2015 [32]	11	Mean 7.7 months (range 6–10)	Retrospective; single IVD; evaluation at 6 months	Significant mean BCVA improvement from 0.58 ± 0.17 logMAR to 0.21 ± 0.15 logMAR and reduction of mean CMT from 513.8 mm to 308.0 mm; no adverse events were noted
6	Sevim et al., 2012 [33]	IVT: 20; PPV: 19	6 months and longer	Retrospective; comparison of BCVA and CMT in two arms: IVT and PPV; evaluation at 12 months	BCVA change at 12 months: IVT: from 0.75 ± 0.23 logMAR to 0.45 ± 0.23 logMAR PPV: 0.78 ± 0.25 logMAR to 0.51 ± 0.21 logMAR; CMT change at 12 months: IVT: 536.00 ± 52.04 mm to 313.15 ± 44.30 mm PPV: 524.05 ± 63.49 mm to 326.31 ± 72.88 mm; significant improvement of BCVA and reduction of CMT at 12 months; no significant difference between the arms at 12 months; temporary
7	Williams et al., 2009 [34]	41	90 days and longer	RCT; CME secondary to uveitis or IGS, persistent 90 days; Three arms IVD (700 mg) or intravitreal dexamethasone 350 mg or observation	Improvement of at least 10 ETDRS letters at day 90: 41.7% in 350 mg group 53.8% in 700 mg group 7.1% in observed group; significant reduction of leakage on FA in treated patients; intraocular pressure rise of 10 mm Hg or more in 5 of 13 patients in the 700 mg group and in 1 of 12 patients in the 350 mg group, controlled by topical medication
8	Koutsandrea et al., 2007 [35]	14	Longer than 6 months	Retrospective; 14 eyes treated with IVT; follow up 12 months	Improvement of BCVA from mean 2.22 ± 0.16 to 0.36 ± 0.24 (decimal values) at 12 months; improvement of BCVA in 11 cases, stable in 2 cases and worsening in 1 case; reduction of CMT from mean 434.93 to 402.79 ± 162.22 mm; reduction of CMT in 11 cases and increase in 3 cases; increase in mf-ERG values; minor increase in IOP; topical IOP-lowering drops in 3 patients

RCT: randomized controlled trial; IGS: Irvine–Gass syndrome; CME: cystoid macular edema; IVD: intravitreal dexamethasone implant (700 mg); IVT: intravitreal triamcinolone; PPV: pars plana vitrectomy; FA: fluorescein angiography; IVB: intravitreal bevacizumab; CMT: central macular thickness; mf-ERG: multifocal electroretinogram; BCVA: best-corrected visual acuity.

**Table 6:** Results of the studies analyzing the efficacy of intravitreal anti-VEGF agents in the treatment of IGS that involved at least 10 cases and were published since 2000.

No	Study	No of Eyes	Duration of CME	Study Design	Results
1	Akay et al., 2020 [38]	59; IVB: 22, IVR: 19, IVA: 18	Not stated; refractory to topical treatment	Retrospective, controlled consecutive case series; comparison of functional and morphological results of treatment among 3 agents at 6 months	BCVA change: IVB: 0.96 ± 0.18 to 0.23 ± 0.19 IVR: 0.89 ± 0.23 to 0.19 ± 0.18 IVA: 0.94 ± 0.22 to 0.21 ± 0.08 CMT change: IVB: 555.5 ± 238.5 mm to 213.5 ± 21.1 mm IVR: 553.5 ± 125.5 mm to 226.6 ± 18.1 mm IVA: 540.0 ± 64.5 mm to 227.7 ± 39.5 mm No of injections: IVB: 1.8 ± 0.7 IVR: 2.0 ± 0.6 IVA: 1.8 ± 0.7 No significant difference in results of treatment and number of injections needed among the three agents
2	Staurenghi et al., 2018 [39]	40	3 months and longer	RCT; IVR 0.5 mg for IGS/aphakic eyes; one injection of IVR at baseline, then PRN regimen	Letter gain at month 2: 8.5 in the IVR group and 4.1 in the sham group (significant difference) At month 12: letter gain 14.5 vs. 10.5; minor adverse events related to injection (e.g., conjunctival hemorrhage)
3	Arevalo et al., 2009 [40]	36	3 months and longer	Retrospective; at least 1 injection of IVB in a dose of 1.25 or 2.5 mg; follow up 12 months	Improvement of BCVA of 2 ETDRS lines in 72.2%; none of the eyes worsened; mean BCVA change from 0.96 to 0.62 logMAR; CMT change from 499.9 to 286.1 mm; Mean no. of injections: 2.7
4	Barone et al., 2009 [41]	10	Mean 17.5 weeks (range 11–24)	At least one IVB 1.25 mg; evaluation of BCVA and CMT at 6 months	BCVA improvement in all eyes; Mean BCVA change from 20/80 to 20/32; mean CMT change from 546.8 to 228.7 mm
5	Spitzer 2008 [42]	16	Mean 14 weeks (range 3–84 weeks)	Retrospective case series; 1.25 mg of IVB; evaluation of BCVA change and CMT change	BCVA improvement by 2 ETDRS letters in 1 eye, unchanged in 12 eyes and worsened in 2 eyes; reduction in CMT by more than 10% in 9 eyes
6	Arevalo et al., 2007 [43]	25	Not stated	Retrospective; IVB of 1.25 or 2.5 mg; mean follow up 32 weeks	Improvement of BCVA of 2 ETDRS lines in 71.4%; none of the eyes worsened; mean BCVA change from 0.92 to 0.50 logMAR; CMT change from 466.3 to 264.5 mm; 28.6% of eyes required a second injection, and 14.3% required a third injection

RCT: randomized controlled trial; IGS: Irvine–Gass syndrome; CME: cystoid macular edema; IVB: intravitreal bevacizumab; IVR: intravitreal ranibizumab; IVA: intravitreal aflibercept; IVD: intravitreal dexamethasone implant (700 mg); BCVA: best-corrected visual acuity; CMT: central macular thickness.

## Data Availability

Not applicable.
